# Effect of Acrylamide Supplementation on the CART-, VAChT-, and nNOS-Immunoreactive Nervous Structures in the Porcine Stomach

**DOI:** 10.3390/ani10040555

**Published:** 2020-03-26

**Authors:** Katarzyna Palus, Michał Bulc, Jarosław Całka

**Affiliations:** Department of Clinical Physiology, Faculty of Veterinary Medicine, University of Warmia and Mazury in Olsztyn, 10-718 Olsztyn, Poland; michal.bulc@uwm.edu.pl (M.B.); calkaj@uwm.edu.pl (J.C.)

**Keywords:** acrylamide, stomach, enteric nervous system, CART, VAChT, nNOS, pig

## Abstract

**Simple Summary:**

The progress of civilization has provided people with virtually unlimited access to food products. However, while the pace of life has increased, the consumption of products with high levels of acrylamide (e.g., chips, corn flakes or coffee) has also increased. The gastrointestinal tract is the first-exposure site for noxious substances ingested with food and it is also often the first defence mechanism. Changes in the expression of neuroactive substances in the intramural neurons of the enteric nervous system (ENS) are a common preclinical symptom of the harmful effect of pathological factors on the body. Using the double immunofluorescence staining method, it was established that supplementation with low and high doses of acrylamide resulted in alterations of the porcine stomach neuron phenotype, which was reflected in an increased number of the cocaine- and amphetamine-regulated transcript (CART)-, vesicular acetylcholine transporter (VAChT)-, and neuronal isoform of nitric oxide synthase (nNOS)-immunoreactive neurons. The recorded changes revealed that even low doses of acrylamide influence the nervous structures located in the porcine gastric wall. This may result from the neurotoxicity of acrylamide or from the response of the ENS to acrylamide-induced inflammation and suggests an important role of the ENS in protecting the gastrointestinal tract during acrylamide intoxication.

**Abstract:**

Acrylamide is found in food products manufactured with high-temperature processing, and exposure to acrylamide contained in food products may cause a potential risk to human health. The aim of this investigation was to demonstrate the changes in the population of CART-, nNOS-, and VAChT-immunoreactive enteric neurons in the porcine stomach in response to supplementation of low and high acrylamide doses. The study was carried out with 15 Danish landrace gilts divided into three experimental groups: the control group—animals were administered empty gelatine capsules; the low-dose group—animals were administrated a tolerable daily intake (TDI) dose (0.5 µg/kg of body weight (b.w.)/day) of acrylamide capsules, and the high-dose group—animals were administrated high-dose (ten times higher than TDI: 5 µg/kg b.w./day) acrylamide capsules for 28 days. Using the double immunofluorescence staining method, it was established that supplementation with low and high doses of acrylamide resulted in alterations of the porcine stomach neuron phenotype, which was reflected in an increased number of CART-, VAChT-, and nNOS-immunoreactive neurons. These changes were accompanied by an increased density of CART-, VAChT-, and nNOS-positive fibres. The results suggest that the enteric nervous system plays an important role in protecting the gastrointestinal tract during acrylamide intoxication.

## 1. Introduction

The oesophageal, gastric, and intestinal wall encompasses a dense network of nerve cells called the enteric nervous system (ENS) [1]. These neurons are organised in the enteric ganglions that are interlinked with a dense network of fibres and are thereby arranged in the plexuses. It is known that in the porcine stomach and in other large animals, as well as in humans, there are two plexuses: the myenteric plexus, situated between the circular and the longitudinal muscular layer, and the submucous plexus located between the tunica muscularis and lamina propria of the mucosa. In the small and large intestines, there are two submucous plexuses: the outer submucous plexus (OSP), located on the inner side of the circular muscular layer, and the inner submucous plexus (ISP), situated near the tunica muscularis in the intestinal mucosa [2,3,4]. It has been demonstrated that the ENS neurons control numerous physiological functions of the gastrointestinal tract (GIT), such as the secretion of digestive fluids, gastric and intestinal motility, and the absorption of nutrients, which are predominantly regulated independently of the central nervous system (CNS) [5]. Each ENS neuron synthesises and secretes a variety of neuroactive substances which are involved in both controlling the physiological functions and in protecting the neurons against the harmful effects of many irritant factors [2,5]. The physiological function of the cocaine- and amphetamine-regulated transcript (CART) in the GIT has not yet been fully elucidated. However, it has been shown that CART enhances gastric acid secretion, causes an inhibitory effect on gastric emptying, and stimulates peristalsis in the colon [6]. The presence of CART in enteric neurons in the porcine stomach, small intestine, and descending colon was described in previous studies [2,7,8]. In contrast, a neuronal isoform of nitric oxide synthase (nNOS) is an enzyme that catalyses the production of nitric oxide (NO) from L-arginine and is considered the major marker of nitrergic neurons. NO is a gaseous inhibitory neurotransmitter. It has a vasodilatory effect, controls the mesenteric blood flow, inhibits intestinal hormone and digestive fluid secretion, and demonstrates a protective effect on the gastric mucosa [9]. Nitric oxide as a non-adrenergic, non-cholinergic inhibitory neurotransmitter detected in different part of the porcine GIT, such as the stomach, duodenum, jejunum, ileum, and descending colon [10,11,12]. In turn, vesicular acetylcholine transporter (VAChT) is believed to be the major marker of cholinergic neurons. It stimulates the secretion of digestive enzymes and intestinal hormones and has a stimulating effect on the gastrointestinal motility [13]. Previous reports confirmed that VACHT-containing cholinergic neurons constitute a significant population of ENS neurons in the porcine GIT. Their presence was noted in the stomach and small intestines [14,15].

Furthermore, numerous studies have demonstrated that the ENS neurons exhibit neuronal plasticity. This kind of plasticity is defined as the adaptation to changes in the external and internal environment, encompassing functional changes, such as a change in the number and transmission of synaptic connections, modification of intracellular signalling cascades, regulation of gene expression, and changes in neurotransmitter secretion [5,16]. Previous studies in animal models and in humans have shown that different pathological conditions in the gastrointestinal tract (inflammation, gastric ulcers, treatment with nonsteroidal anti-inflammatory drugs (NSAIDs), diabetes mellitus, mycotoxins in feed or bisphenol-A intoxication) changed the neurochemical profile of the enteric neurons and had a significant effect on modifying the number of CART-, nNOS- or VAChT-immunoreactive neurons [2,7,10,11,17,18,19].

Acrylamide (ACM) (an α, β-unsaturated reactive monomer) is used in many industrial branches, such as polyacrylamide production, the manufacturing of beauty products, glues, paper, and polyacrylamide gels [20]. ACM is known to have carcinogenic and genotoxic properties, a harmful effect on reproduction, and is said to be neurotoxic [20,21]. Among the many adverse effects of acrylamide, only its neurotoxic effects have been documented in humans [21]. However, only after discovering that ACM is found in food products manufactured with high-temperature processing was a series of studies conducted to investigate food-related exposure as a potential risk to human health [22]. ACM is synthesized in the Maillard reaction between L-asparagine and reducing sugars. A very high level of acrylamide is found in potato chips, French fries, corn flakes, crackers, and coffee, all food products which are very popular among young consumers [23]. Epidemiological studies have demonstrated that the average acrylamide intake in adults varies between 0.3 and 0.8 μg/kg body weight (b.w.) per day [24]. The maximum safe intake levels have not yet been determined for acrylamide found in food products. Despite numerous studies and confirmation that the gastrointestinal tract is the main absorption route of ACM, little is known about its effect on the ENS neurons.

The aim of this study was to demonstrate the changes in the population of CART-, nNOS-, and VAChT-immunoreactive enteric neurons in the porcine stomach in response to supplementation of low and high acrylamide doses. The study was conducted on pigs which (as an omnivorous species) have similar anatomy and physiological processes to humans [25]. For years, pigs have been used in biomedical research, especially in studies investigating the gastrointestinal system [26].

## 2. Materials and Methods

**Figure 1:**
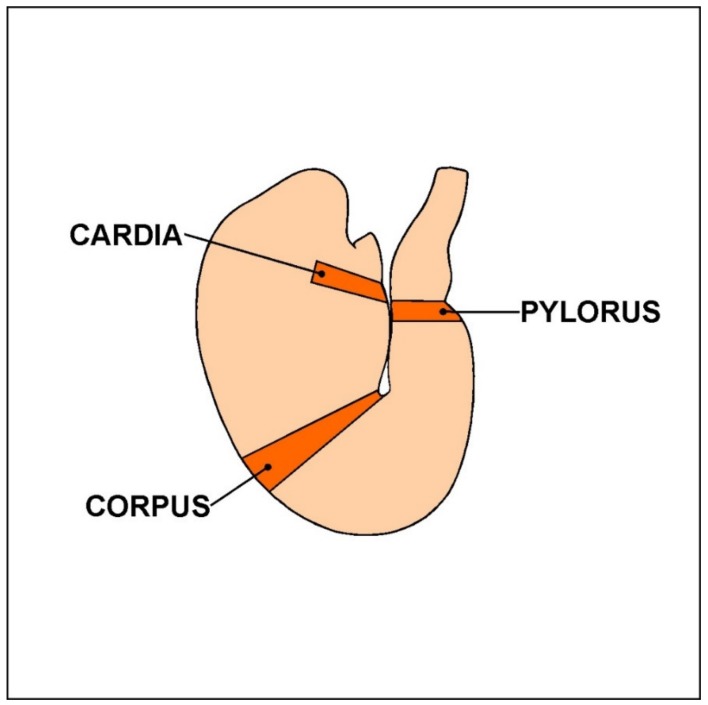
The porcine stomach.

The study was carried out with 15 Danish landrace gilts with an approximate body weight of 15 kg, originating from a farm. The animals were kept in common pens suitable for their age, were provided water ad libitum, and were fed a commercial pig feed mixture. After a one-week acclimatization period, the gilts were divided into three experimental groups: 1. control group (C group, n = 5): the animals were administered empty gelatine capsules; 2. low-dose group (LD group, n = 5): the animals were administrated a tolerable daily intake (TDI) dose (0.5 µg/kg b.w./day) of acrylamide (>99%; Sigma–Aldrich, Poznań, Poland) capsules; 3. high-dose group (HD group, n = 5): the animals were administrated high-dose (ten times higher than TDI: 5 µg/kg b.w./day) acrylamide capsules. All experimental procedures were approved by the Local Ethical Committee for Experiments on Animals in Olsztyn (Approval No. 11/2017). In each group, the animals were administered the specific capsules with morning feeding for four weeks. After a 28-day treatment, all gilts were euthanized with a lethal anaesthetic injection (sodium pentobarbital, Morbital, Biowet Puławy, Puławy, Poland), and stomach samples from the cardia, corpus, and pylorus region (Figure 1) were immediately collected and fixed by immersion in a 4% buffered solution of paraformaldehyde (pH = 7.4) for 1 h and then placed in a phosphate buffer solution (PBS, pH 7.4) for three days (the buffer was changed daily). Finally, samples were placed into an 18% buffered solution of sucrose (pH 7.4) for two weeks.

The illustration shows the porcine stomach with marked fragments collected for further study.

Frozen sections (14 µm thick) from the collected stomach samples were then processed with the double immunofluorescent staining method (as described previously by Palus et al. [27]). On day 1, the sections were dried at room temperature, rinsed three times in PBS (10 min), and blocked with the blocking mixture (10% horse serum, 0.1% bovine serum albumin in 0.1 M PBS, 1% Triton X-100, 0.05% thimerosal, and 0.01% sodium azide) for 1 h, rinsed again three times in PBS (10 min). The primary antibody solution was then added (Table 1), and the sections were incubated overnight in a humid chamber. On day 2, the sections were rinsed three times in PBS (10 min) and incubated with the secondary antibody solution (Table 1) for one hour, and after three rinsing steps in PBS (10 min), the tissue sections were immersed in a glycerol solution and covered with a coverslip. As a negative control, the following tests were used: pre-absorption for antisera with appropriate antigens and an omission and replacement test. No fluorescence was observed in any of the tests.

After staining, the sections were examined with an Olympus BX51 fluorescent microscope. To determine the number of individual neuron populations, a minimum of 500 PGP 9.5-positive neurons were analysed for each of the investigated neuroactive substances in both types of the plexuses (myenteric and submucous), and the number of PGP 9.5-positive neurons was assumed as 100%. The sections were separated by a distance of at least 200 µm to avoid counting the same neurons. To estimate the density of nerve fibres immunoreactive to CART, nNOS, and VAChT in the circular muscle layer (CML) and the submucosal/mucosal layer (S/ML), an arbitrary scale was used, in which (-) indicated a lack of fibres immunoreactive to CART, nNOS or VACHT, and (+++) indicated a dense network of nerve fibres. The results were statistically processed with Statistica 12 software (StatSoft Inc., Tulsa, OK, USA) and were expressed as the mean ± standard error of the mean (SEM). Statistically significant differences were evaluated with one-way analysis of variance (ANOVA) with Dunnett’s test (* *p* < 0.05, ** *p* < 0.01, *** *p* < 0.001).

## 3. Results

### 3.1. Myenteric Plexus

**Figure 2:**
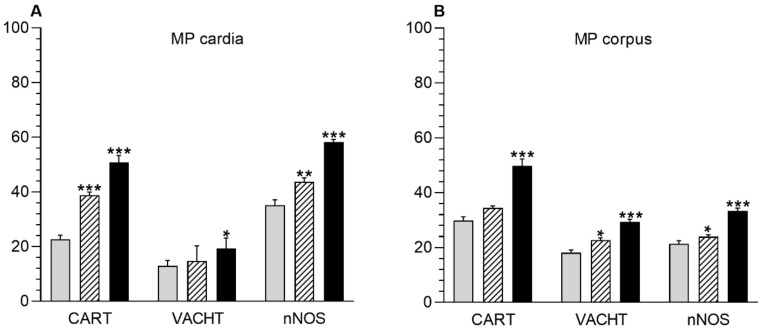

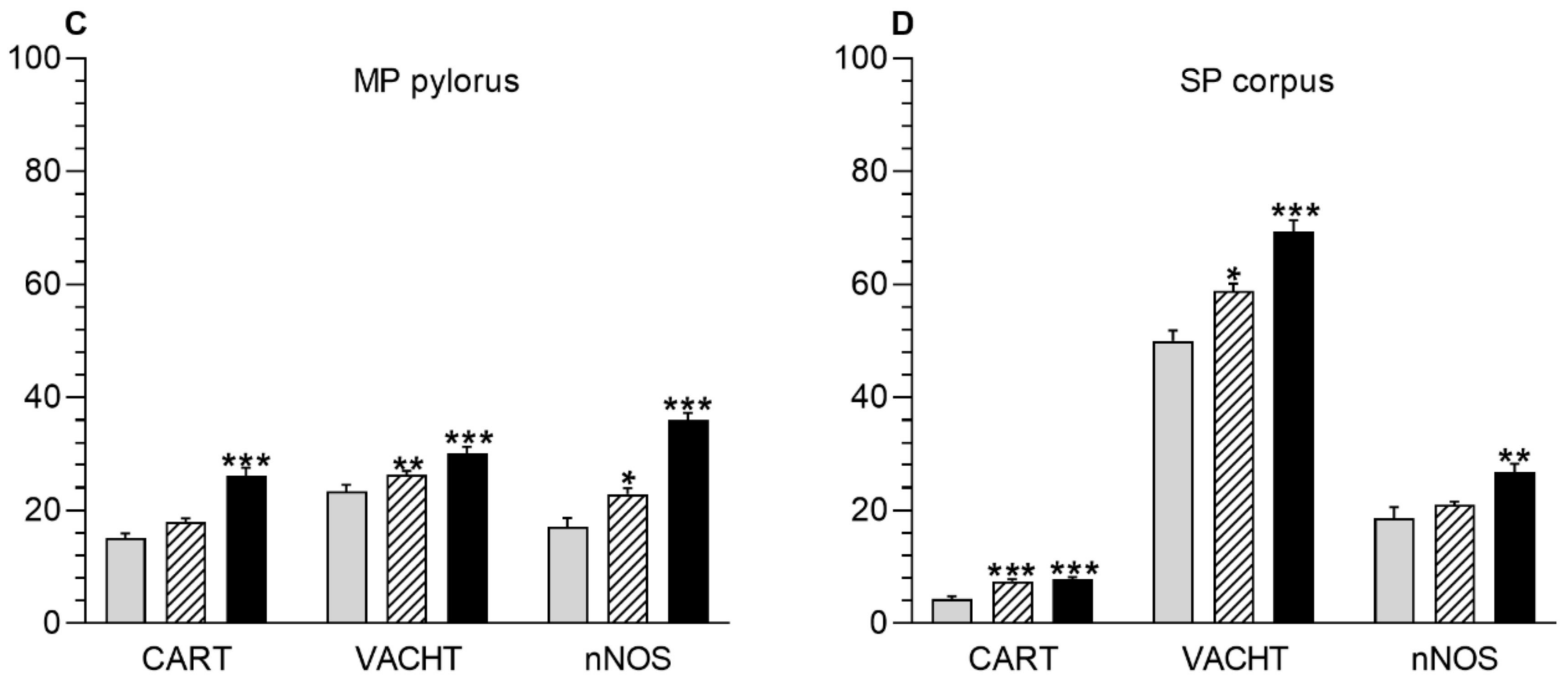
Acrylamide-induced changes in number of stomach enteric neurons immunoreactive to the cocaine- and amphetamine-regulated transcript (CART), the vesicular acetylcholine transporter (VAChT) and the neuronal isoform of nitric oxide synthase (nNOS). Enteric neurons immunoreactive to CART, VAChT, and nNOS in the myenteric plexuses (MP) of cardia (**A**), corpus (**B**), and pylorus (**C**) and in the submucous plexuses (SP) of corpus (**D**) in animals from the control (grey bar), LD (lined bar), and HD (black bar) groups. * *p* < 0.05,** *p* < 0.01,*** *p* < 0.001 indicate differences in the expression of particular substance studied in comparison to the control animals.

**Figure 3:**
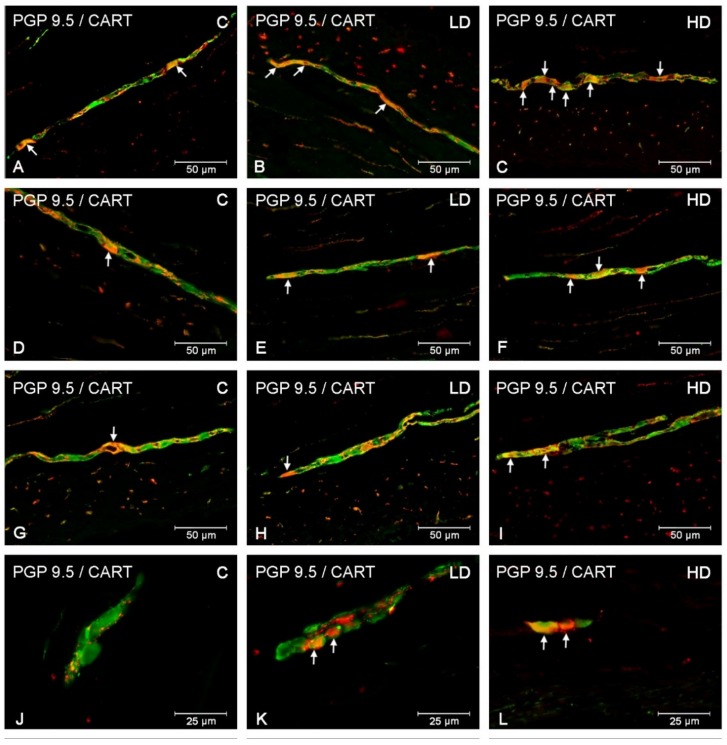
CART-immunoreactive neurons in the porcine stomach. The pictures show the cocaine- and amphetamine-regulated transcript (CART)-immunoreactive intramural neurons in the wall of the porcine stomach in the control group and in animals receiving low and high doses of acrylamide. (**A**)—myenteric neurons immunoreactive to protein gene-product 9.5 (PGP 9.5), used as a pan-neuronal marker, and CART in the cardia of control animals; (**B**)—myenteric neurons immunoreactive to PGP 9.5 and CART in the cardia of animals from LD group; (**C**)—myenteric neurons immunoreactive to PGP 9.5 and CART in the cardia of animals from HD group; (**D**)—myenteric neurons immunoreactive to PGP 9.5 and CART in the corpus of control animals; (**E**)—myenteric neurons immunoreactive to PGP 9.5 and CART in the corpus of animals from LD group; (**F**)—myenteric neurons immunoreactive to PGP 9.5 and CART in the corpus of animals from HD group; (**G**)—myenteric neurons immunoreactive to PGP 9.5 and CART in the pylorus of control animals; (**H**)—myenteric neurons immunoreactive to PGP 9.5 and CART in the pylorus of animals from LD group; (**I**)—myenteric neurons immunoreactive to PGP 9.5 and CART in the pylorus of animals from HD group; (**J**)—submucous neurons immunoreactive to PGP 9.5 and CART in the corpus of control animals; (**K**)—submucous neurons immunoreactive to PGP 9.5 and CART in the corpus of animals from LD group; (**L)**—submucous neurons immunoreactive to PGP 9.5 and CART in the corpus of animals from HD group.

**Figure 4:**
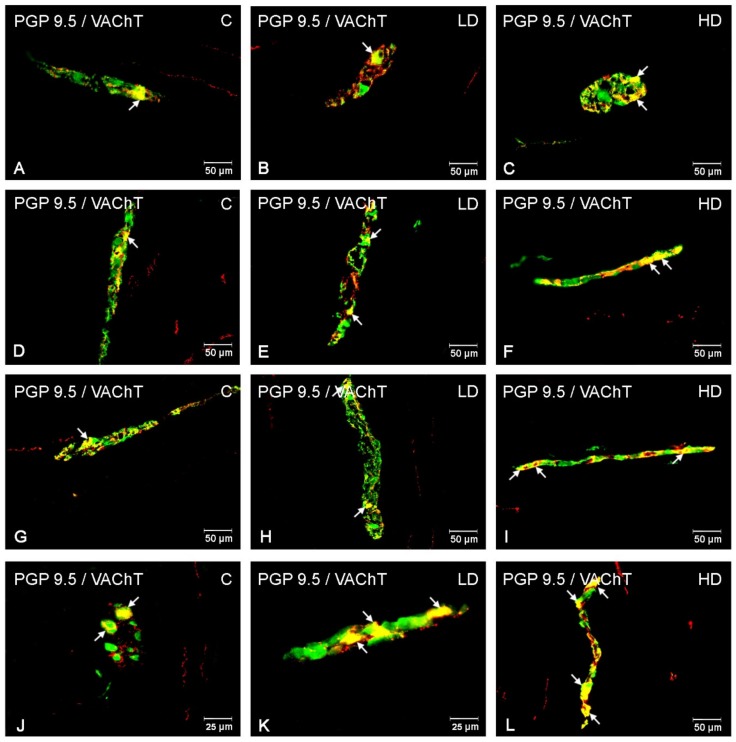
VAChT-immunoreactive neurons in the porcine stomach. The pictures show the vesicular acetylcholine transporter (VAChT)-immunoreactive intramural neurons in the wall of the porcine stomach in the control group and in animals receiving low and high doses of acrylamide. (**A**)—myenteric neurons immunoreactive to protein gene-product 9.5 (PGP 9.5), used as a pan-neuronal marker, and VAChT in the cardia of control animals; (**B**)—myenteric neurons immunoreactive to PGP 9.5 and VAChT in the cardia of animals from LD group; (**C**)—myenteric neurons immunoreactive to PGP 9.5 and VAChT in the cardia of animals from HD group; (**D**)—myenteric neurons immunoreactive to PGP 9.5 and VAChT in the corpus of control animals; (**E**)—myenteric neurons immunoreactive to PGP 9.5 and VAChT in the corpus of animals from LD group; (**F**)—myenteric neurons immunoreactive to PGP 9.5 and VAChT in the corpus of animals from HD group; (**G**)—myenteric neurons immunoreactive to PGP 9.5 and VAChT in the pylorus of control animals; (**H**)—myenteric neurons immunoreactive to PGP 9.5 and VAChT in the pylorus of animals from LD group; (**I**)—myenteric neurons immunoreactive to PGP 9.5 and VAChT in the pylorus of animals from HD group; (**J**)—submucous neurons immunoreactive to PGP 9.5 and VAChT in the corpus of control animals; (**K**)—submucous neurons immunoreactive to PGP 9.5 and VAChT in the corpus of animals from LD group; (**L**)—submucous neurons immunoreactive to PGP 9.5 and VAChT in the corpus of animals from HD group.

**Figure 5:**
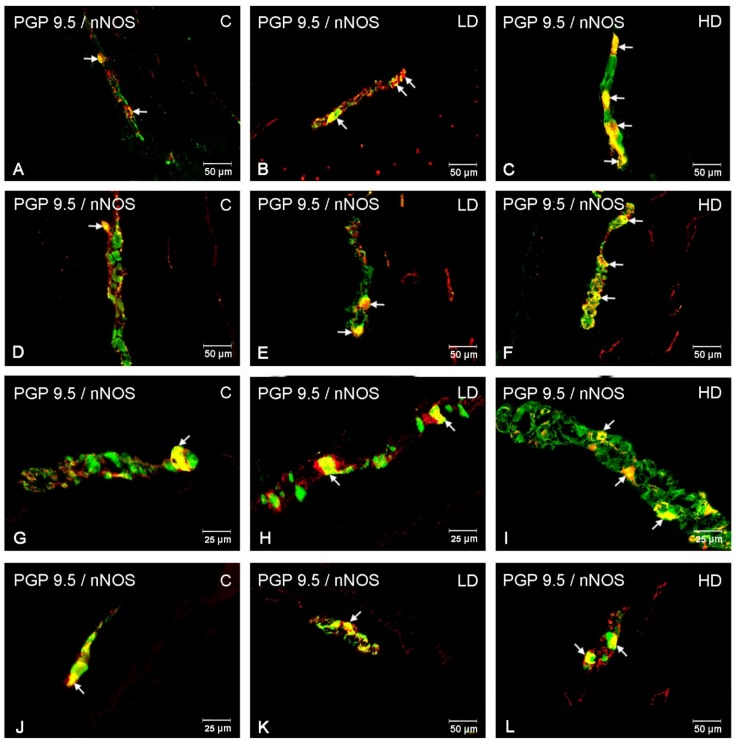
nNOS-immunoreactive neurons in the porcine stomach. The pictures show the neuronal isoform of nitric oxide synthase (nNOS)- immunoreactive intramural neurons in the wall of the porcine stomach in the control group and in animals receiving low and high doses of acrylamide. (**A**)—myenteric neurons immunoreactive to protein gene-product 9.5 (PGP 9.5), used as a pan-neuronal marker, and nNOS in the cardia of control animals; (**B**)—myenteric neurons immunoreactive to PGP 9.5 and nNOS in the cardia of animals from LD group; (**C**)—myenteric neurons immunoreactive to PGP 9.5 and nNOS in the cardia of animals from HD group; (**D**)—myenteric neurons immunoreactive to PGP 9.5 and nNOS in the corpus of control animals; (**E**)—myenteric neurons immunoreactive to PGP 9.5 and nNOS in the corpus of animals from LD group; (**F**)—myenteric neurons immunoreactive to PGP 9.5 and nNOS in the corpus of animals from HD group; (**G**)—myenteric neurons immunoreactive to PGP 9.5 and nNOS in the pylorus of control animals; (**H**)—myenteric neurons immunoreactive to PGP 9.5 and nNOS in the pylorus of animals from LD group; (**I**)—myenteric neurons immunoreactive to PGP 9.5 and nNOS in the pylorus of animals from HD group; (**J**)—submucous neurons immunoreactive to PGP 9.5 and nNOS in the corpus of control animals; (**K**)—submucous neurons immunoreactive to PGP 9.5 and nNOS in the corpus of animals from LD group; (**L**)—submucous neurons immunoreactive to PGP 9.5 and nNOS in the corpus of animals from HD group.

In the myenteric plexus, the administration of acrylamide increased the CART-, VAChT-, and nNOS-immunoreactive (IR) neuronal populations in all investigated stomach fragments (Figure 2). For CART, the major changes were found in the cardia, i.e., an increase from 22.72 ± 1.45% to 38.75 ± 1.18% and to 50.73 ± 2.61% in the LD and HD groups, respectively (Figure 2A, Figure 3A–C). In the stomach corpus region, the changes were also significant for low-dose and high-dose acrylamide supplementation (an increase from 29.86 ± 1.35% to 34.45 ± 0.72% and 49.80 ± 2.47%, respectively) (Figure 2B, Figure 3D–F). The smallest changes were demonstrated in the pylorus, where the number of CART-immunoreactive (CART-IR) neurons increased only in the HD group (from 15.15 ± 0.80% to 26.16 ± 1.39%) (Figure 2C, Figure 3G,I). In contrast, for VAChT, the greatest changes were found in the pylorus, where the number of VAChT-IR neurons increased from 23.41 ± 1.11% in the control group to 26.32 ± 0.69% in the LD group and 30.08 ± 1.23% in the HD group (Figure 2C, Figure 4G–I). In the corpus, the changes were statistically significant in both experimental groups (an increase from 18.10 ± 1.01% to 22.73 ± 0.86% and 29.42 ± 0.87%) (Figure 2B, Figure 4D–F), whereas in the cardia, a substantial increase in the VAChT-IR neuronal population (from 13.01 ± 0.87% to 19.23 ± 1.23%) was only found in the HD group (Figure 2A, Figure 4A,C). In the nNOS case, the greatest changes were demonstrated in the cardia, where the number of nNOS- immunoreactive (NOS-IR) neurons increased from 35.27 ± 1.85% to 43.69 ± 1.47% in the LD group and to 58.21 ± 1.04% in the HD group (Figure 2A, Figure 5A–C). A slightly smaller alteration was recorded in the pylorus: from 17.16 ± 1.53% in the control group to 22.89 ± 1.06% and to 36.03 ± 1.23% in the experimental groups (Figure 2C, Figure 5G–I). The smallest increase was detected in the corpus, as statistically significant differences were demonstrated only in the group supplemented with high acrylamide doses (an increase from 21.38 ± 1.10% to 33.36 ± 1.06%) (Figure 2D, Figure 5D,F).

### 3.2. Submucous Plexus

Acrylamide also induced a substantial change in the CART-, VACHT-, and nNOS-IR neuronal populations in the submucous plexuses in the stomach corpus (Figure 2). For CART, a highly significant increase in the number of CART-IR neurons was found in the LD group (from 4.32 ± 0.53% to 7.52 ± 0.36%) and HD group (to 7.76 ± 0.42%) (Figure 2D, Figure 3J–L). An increase in the VAChT-IR neurons was also recorded in both experimental groups: from 50.05 ± 1.83% to 58.81 ± 1.38% and to 69.39 ± 1.95%, respectively (Figure 2D, Figure 4J–L). While for nNOS, a statistically significant increase in the nNOS-IR neurons was shown only in the HD group (from 18.64 ± 1.96% to 26.87 ± 1.37%) (Figure 2D, Figure 5J,L). In the submucous plexuses of the cardia and pylorus region, no neurons immunoreactive to the investigated neuroactive substances were found.

### 3.3. Nerve Fibres

**Figure 6:**
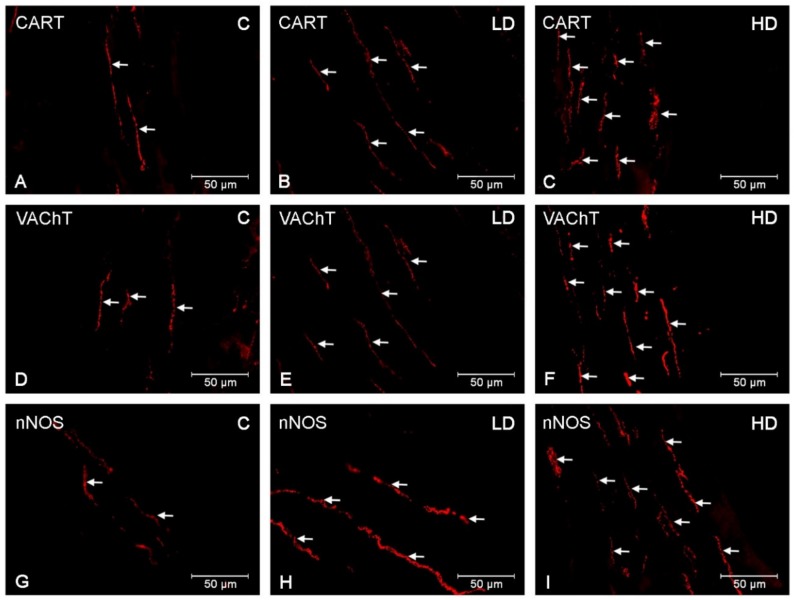
Nerve fibres in the circular muscle layer. The pictures show nerve fibres immunoreactive to the the cocaine- and amphetamine-regulated transcript (CART), the vesicular acetylcholine transporter (VAChT) and the neuronal isoform of nitric oxide synthase (nNOS) in the circular muscle layer (CML) in the porcine stomach in the control group and in animals receiving low and high doses of acrylamide. (**A**)—CART-immunoreactive nerve fibres in the CML in the corpus of control animals; (**B**)—CART-immunoreactive nerve fibres in the CML in the corpus of animals from LD group; (**C**)—CART-immunoreactive nerve fibres in the CML in the corpus of animals from HD group; (**D**)—VAChT-immunoreactive nerve fibres in the CML in the pylorus of control animals, (**E**)—VAChT-immunoreactive nerve fibres in the CML in the pylorus of animals from LD group, (**F**)—VAChT-immunoreactive nerve fibres in the CML in the pylorus of animals from HD group, (**G**)—nNOS-immunoreactive nerve fibres in the CML in the pylorus of control animals, (**H**)—nNOS-immunoreactive nerve fibres in the CML in the pylorus of animals from LD group, (**I**)—nNOS-immunoreactive nerve fibres in the CML in the pylorus of animals from HD group.

**Figure 7:**
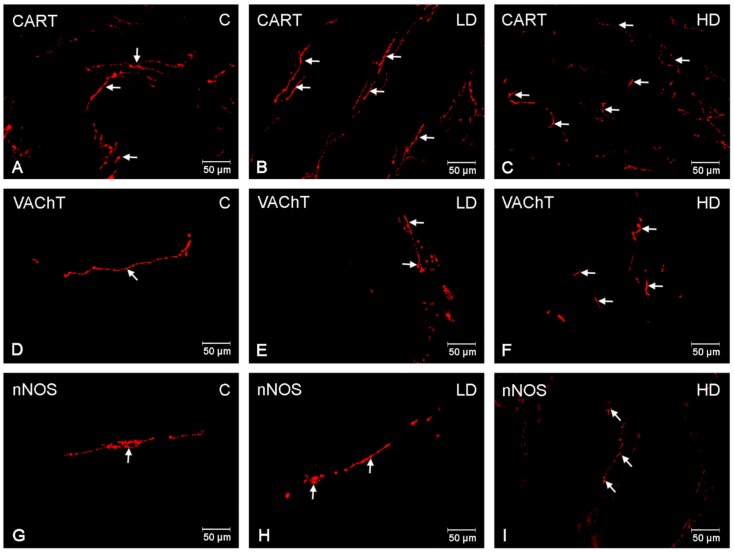
Nerve fibres in the submucous/mucous layer. The pictures show nerve fibres immunoreactive to the the cocaine- and amphetamine-regulated transcript (CART), the vesicular acetylcholine transporter (VAChT) and the neuronal isoform of nitric oxide synthase (nNOS in the submucous/mucous layer (S/ML) in the porcine stomach in the control group and in animals receiving low and high doses of acrylamide. (**A**)—CART-immunoreactive nerve fibres in the S/ML in the corpus of control animals; (**B**)—CART-immunoreactive nerve fibres in the S/ML in the corpus of animals from LD group; (**C**)—CART-immunoreactive nerve fibres in the S/ML in the corpus of animals from HD group; (**D**)—VAChT-immunoreactive nerve fibres in the S/ML in the corpus of control animals, (**E**)—VAChT-immunoreactive nerve fibres in the S/ML in the corpus of animals from LD group, (**F**)—VAChT-immunoreactive nerve fibres in the S/ML in the corpus of animals from HD group, (**G**)—nNOS-immunoreactive nerve fibres in the S/ML in the pylorus of control animals, (**H**)—nNOS-immunoreactive nerve fibres in the S/ML in the pylorus of animals from LD group, (**I**)—nNOS-immunoreactive nerve fibres in the S/ML in the pylorus of animals from HD group.

CART-, VAChT-, and nNOS-immunoreactive nervous fibres were detected in both the circular muscle layer (CML) and the submucosal/mucosal layer (S/ML) in all investigated gastric regions (Table 2). In the CML, a dense network of CART-IR nerve fibres was demonstrated in the cardia (++), corpus (+++) (Figure 6A) and pylorus (++). Fibres with slightly smaller density were found in the S/ML (+/++) (Figure 7A), although few varicose VACHT (+) and nNOS (+) fibres were detected in the control group or in the CLM (Figure 6D,G) and the S/ML (Figure 7D,G). The VACHT- and nNOS-immunoreactive fibre density was comparable in all investigated gastric regions (cardia, corpus, and pylorus). Acrylamide produced a substantial increase in the CART-, VAChT-, and nNOS-immunoreactive fibre density in both the CML (Figure 6B,C,E,F,H,I) and S/ML (Figure 7B,C,E,F,H,I). The greatest changes were demonstrated in the HD acrylamide group; however, even in the LD group, changes were also noticeable (Table 2).

## 4. Discussion

The progress of civilization and the development of industry have provided people with virtually unlimited access to food products. However, while the pace of life has increased, the consumption of products with high levels of acrylamide is also on the rise. The gastrointestinal tract is the first-exposure site to noxious substances ingested with food, and it is also often the first defence mechanism. Changes in the expression of neuroactive substances in the intramural neurons of the ENS are a common preclinical symptom of the harmful effect of pathological factors on the body. The investigation showed that the supplementation of both low and high doses of acrylamide had a substantial effect on the population of the enteric CART-, VAChT-, and nNOS-immunoreactive neurons and the fibre density in the porcine stomach. The degree of these changes was variable, depending on the specific stomach region and the type of investigated plexus. Nevertheless, the findings may also indicate that the alimentary exposure to acrylamide causes significant alterations in the neurochemical phenotype of the enteric neurons and is therefore not neutral in the body.

Acrylamide is categorised as a neurotoxic compound, with toxic effects on the central nervous system (CNS) and peripheral nervous system (PNS), both in laboratory animals and in humans [28]. The reported symptoms of acrylamide exposure include limb numbness, muscle weakness or ataxia, and they most probably result from an inhibition of the kinesin-based fast axonal transport and alterations in the neurotransmitter levels [29,30,31]. Acrylamide binds with protein receptors rich in cysteine that are involved in the presynaptic release of the neurotransmitters, membrane reuptake, and vesicular nerve conduction, which results in disturbed synaptic transmission [32]. It has also been demonstrated that acrylamide causes damage to nerve ends and Purkinje cells, as well as axonal oedema [33]. In acrylamide intoxication, an increase in dopamine expression has also been shown in the rat striatum [34]. Furthermore, acrylamide results in a reduction of enzymatic and non-enzymatic antioxidants and in lipid peroxidation, which leads to nerve cell apoptosis, and the latter phenomenon is involved in the pathogenesis of many neurodegenerative diseases [35,36]. This has also been demonstrated in some studies on acrylamide-induced reactive gliosis, presenting with a potentiated synthesis of free radicals and stimulating amino acids and proinflammatory cytokines in the glial cells, which results in neuronal death [35]. Previous studies have not elucidated how acrylamide impacts the enteric neurons. However, the above-mentioned studies on different parts of the nervous system may indicate that these findings are a result of neurotoxicity.

Changes in neurotransmitter expression in the enteric neurons are a result of adaptation to irritants to which the cell is exposed, and it should accommodate the neurons to survive under variable, often unfavourable conditions. Previous studies investigating the ENS have shown that many of the neuroactive substances synthesized by these neurons have neuroprotective properties [11,16,18]. Undoubtedly, CART is one of them. In the investigation, the reported increase in the CART-IR neuron population is consistent with previous studies that demonstrated an elevated CART expression in the enteric neurons due to mycotoxin intoxication, neuron damage, diabetes or hypertension [2,7,37,38]. The severity of these alterations was determined by the section of the gastrointestinal (GI) tract and the given pathology. The neuroprotective effect of CART has been also evidenced in in vitro mice studies since it found increased survival of neurons in a CART-supplemented myenteric neuron culture [39]. Acetylcholine (ACh) is also an important endogenous neurotransmitter, and its neuroprotective effects have been demonstrated in both the central and peripheral nervous systems [40,41]. Recent studies have found that the population of neurons immunoreactive to VAChT (a marker of the cholinergic neurons) in the enteric neuronal population increased during irinotecan treatment [42]. The elevated number of VAChT-immunoreactive neurons reported in the presented investigation is also consistent with previous studies that demonstrated an elevation of acetylcholinesterase (AChE) levels in the peripheral cholinergic neurons during acrylamide intoxication [43]. Interestingly, VAChT is also believed to be an excellent morphological indicator to investigate the regenerative process of motor neuron endings [44]. In turn, nNOS may present a two-pronged effect, depending on the site of a pathological process in the GI tract and the nature of the noxious stimulus. An elevated number of intramural nNOS-immunoreactive neurons has been reported in many GI dysfunctions, such as hyperacidity of the stomach and naproxen treatment [12,18]. Furthermore, it has been shown that the levels of transcription factors involved in protecting the myenteric neurons against ischaemia–reperfusion injury increased, and the survival of the rat myenteric neurons in an nNOS-supplemented culture was elevated, which provides evidence of the neuroprotective effect of NO [45,46]. There are also reports on the reduced expression of nNOS in enteric neurons, for example, in diabetes or Crohn disease [10,47]. However, they prove the above-mentioned bidirectionality of NO effects in response to different neurotoxic stimuli.

Although the neurotoxicity of acrylamide has been well discussed, it cannot be precluded that the changes in the neurochemical phenotype of the stomach enteric neurons, reported in the present study, may result from a response to inflammation. It has been shown that acrylamide has proinflammatory properties. There is some evidence that acrylamide increases interleukin-6 (Il-6) and tumour necrosis factor α (TNF-α) levels in the blood serum via overproduction of reactive oxygen species (ROS) and therefore leads to neuroinflammation [35]. Previous studies conducted by the authors have also demonstrated that supplementation with acrylamide results in local ileitis with such symptoms as the increased synthesis of proinflammatory cytokines by gut-associated lymphoid tissue (GALT) (Interleukin 1β (IL-1β), IL-6, TNF-α) [48]. The authors’ findings correlate with the previous studies in which an elevated number of CART-, VAChT-, and nNOS-IR neurons was detected in the course of different inflammatory GIT conditions. In particular, an increased immunoreactivity to CART in the ENS structures was recorded in ulcerative colitis and experimentally induced colitis in pigs [38,49]. It has been proven that the cholinergic system is involved in the immune response mechanisms by stimulating the nicotine receptors and thereby leading to a reduction in the inflammatory response in the CNS [50]. Additionally, it decreases lipid peroxidation by removing reactive oxygen species (ROS) [51]. A direct inhibitory effect of ACh on the synthesis of proinflammatory cytokines by macrophages has been demonstrated [52]. NO is known for its anti-inflammatory and proinflammatory properties in the gastrointestinal tract. The outcome depends on the type of inflammation and its site. Nevertheless, it has been shown that NO is involved in protecting the gastric mucosa against damage, supporting ulcer healing, and increasing cytokine synthesis in the mucosa [53,54,55]. Considering these data, it may be speculated that the investigated neuroactive substances are involved in controlling acrylamide-induced inflammation in the porcine stomach.

It is also acknowledged that despite the lack of data on the toxic mechanism of acrylamide in the ENS structures, the demonstrated changes may result from an elevated synthesis of the investigated neuroactive substances on its different stages, such as transcription, translation or modification of the activity of enzymes involved in this synthesis, or these changes may be a consequence of the inhibition of axonal transport or slowed degradation of neurotransmitters. However, the reported increased density of the CART-, VAChT-, and nNOS-positive fibres associated with the elevated number of neurons, which are immunoreactive to the investigated substances, supports the increased synthesis scenario.

## 5. Conclusions

To conclude, the discussed investigation has demonstrated that supplementation with low and high doses of acrylamide resulted in alterations of the porcine stomach neuron phenotype, which was reflected in an increased number of CART-, VAChT-, and nNOS-immunoreactive neurons. These changes were accompanied by an increased density of CART-, VAChT-, and nNOS-positive fibres. The recorded changes revealed that even low doses of acrylamide influenced the nervous structures located in the porcine gastric wall. This may result from the neurotoxicity of acrylamide or from the response of the ENS to acrylamide-induced inflammation. The authors’ studies also suggest that the ENS plays an important role in protecting the gastrointestinal tract during acrylamide intoxication. Considering the role of the pig as an important model in biomedical research, these results may become a topic of further toxicological and clinical studies on reducing the harmful effects of acrylamide found in food products in the body.

## Figures and Tables

**Table 1:** List of immunoreagents used in the present study.

Antigen	Host Species	Code	Dilution	Manufacturer/Supplier
**Primary antibodies**				
**PGP 9.5**	Mouse	7863-2004	1:1000	Bio-Rad, Hercules, CA, USA
**CART**	Rabbit	H-003-61	1:16000	Phoenix Pharmaceuticals, Burlingame, CA, USA
**VAChT**	Rabbit	H-V007	1:2000	Phoenix Pharmaceuticals, Burlingame, CA, USA
**nNOS**	Rabbit	AB5380	1:2000	Sigma–Aldrich, Saint Louis, MO, USA
**Secondary antibodies**				
**Alexa Fluor 488 nm donkey anti-mouse IgG**		A21202	1:1000	Thermo Fisher Scientific, Waltham, MA, USA
**Alexa Fluor 546 nm goat anti-rabbit IgG**		A11010	1:1000	Thermo Fisher Scientific, Waltham, MA, USA

PGP 9.5-protein gene product 9.5, CART- the cocaine- and amphetamine-regulated transcript, nNOS- the neuronal isoform of nitric oxide synthase, the cocaine vesicular acetylcholine transporter (VAChT).

**Table 2:** Density of nerve fibres immunoreactive to the cocaine- and amphetamine-regulated transcript (CART), the vesicular acetylcholine transporter (VAChT) and the neuronal isoform of nitric oxide synthase (nNOS) in the stomach wall.

Part of the Stomach	Cardia			Corpus			Pylorus		
	C Group	LD Group	HD Group	C Group	LD Group	HD Group	C Group	LD Group	HD Group
CART									
CML	++	++	+++	+++	+++	++++	++	++	+++
S/ML	+	+	++	+	++	+++	++	++	+++
VACHT									
CML	+	+	++	+	+	++	+	++	++
S/ML	+	+	+	+	++	++	+	++	++
nNOS									
CML	+	+	++	+	++	++	+	++	+++
S/ML	+	+	++	+	+	++	+	++	++

CML-the circular muscle layer, S/ML-the submucous/mucous layer.
